# Beneficial Effects of a Low-dose of Conjugated Linoleic Acid on Body Weight Gain and other Cardiometabolic Risk Factors in Cafeteria Diet-fed Rats

**DOI:** 10.3390/nu12020408

**Published:** 2020-02-04

**Authors:** Miguel Z Martín-González, Héctor Palacios, Miguel A Rodríguez, Lluís Arola, Gerard Aragonès, Begoña Muguerza

**Affiliations:** 1 Department of Biochemistry and Biotechnology, Nutrigenomics Research Group, Universitat Rovira i Virgili, 43007 Tarragona, Spain; miguelmg.1991@gmail.com (M.Z.M.-G.); hector.palacios@eurecat.org (H.P.); lluis.arola@urv.cat (L.A.); begona.muguerza@urv.cat (B.M.); 2 Eurecat, Centre Tecnològic de Catalunya, Centre for Omic Sciences (COS), Joint Unit Universitat Rovira i Virgili-EURECAT, Unique Scientific and Technical Infrastructures (ICTS), 43204 Reus, Spain; miguelangel.rodriguez@eurecat.org; 3 Eurecat, Centre Tecnològic de Catalunya, Biotechnological Area, 43204 Reus, Spain

**Keywords:** CLA, insulin resistance, leptin, metabolomics, NAFLD, obesity

## Abstract

Conjugated linoleic acid (CLA) is a dietary supplement that has been shown to improve obesity. However, some authors have associated high doses of CLA supplementation with liver impairment and insulin resistance. The aim of this study was to assess whether the consumption of low doses of CLA maintained the beneficial effects on the main metabolic disturbances associated with metabolic syndrome (MetS) but prevented the occurrence of non-desirable outcomes associated with its consumption. Male Wistar rats, fed standard or cafeteria (CAF) diet for 12 weeks, were supplemented with three different low doses of CLA in the last three weeks. Both biochemical and H^1^ NMR-based metabolomics profiles were analysed in serum and liver. The consumption of 100 mg/kg CLA, but not doses of 200 and 300 mg/kg, ameliorated the increase in body weight gain as well as the serum concentrations of glucose, insulin, cholesterol, triglyceride, diglyceride, and total phospholipid induced by a CAF diet. In turn, CLA reverted the increase in lactate, alanine, and glucose concentrations in the liver of these animals, but enhanced hepatic cholesterol accumulation without any detrimental effect on liver function. In conclusion, a low dose of CLA corrected the adverse effects associated with MetS without compromising other metabolic parameters.

## 1. Introduction

The obesity epidemic is a major health risk factor that frequently occurs concurrently with many other cardiovascular risk factors related to lifestyle, such as dyslipidaemia, impaired glucose tolerance or non-alcoholic fatty liver disease (NAFLD), resulting in metabolic syndrome (MetS) [1]. This disease is considered as such when at least three of the following five symptoms are present: high waist circumference, hypertriglyceridaemia, hyperglycaemia, hypertension, and reduced high-density lipoprotein (HDL) cholesterol concentrations. These symptoms provide an easy-to-assess method of diagnosing MetS, but do not provide a full picture of the underlying problem. Metabolomics studies have found many links between MetS and particular metabolites that could be used to better characterise this disease [2]. In this sense, Cheng et al. studied the plasma concentrations of 45 metabolites in the Framingham Heart Study (*n* = 1015) and the Malmö Diet and Cancer Study (*n* = 746). Metabolic risk factors such as obesity, insulin resistance, hypertension and dyslipidaemia were associated with multiple metabolites including branched-chain amino acids (BCAAs), other hydrophobic amino acids, tryptophan breakdown products, and nucleotide metabolites [3]. Other studies have also reported a strong association between plasma metabolites and cardiometabolic risk factors such as increased levels of BCAAs and aromatic amino acids with increased proinflammatory mediators and metabolic disease [4], high alanine levels with insulin resistance [2], and low levels of histidine with inflammation, oxidative stress, and mortality in patients which chronic kidney disease [5]. In addition, reduced plasma levels of lysine and methionine have been pointed to as important contributors and early biomarkers of incipient MetS [4]. Other metabolites such as the phospholipids phosphatidylcholine and phosphatidylethanolamine have also emerged as biomarkers that correlated with features of MetS, as well as adipose tissue dysfunction and inflammation [6]. 

Wistar rats fed a cafeteria (CAF) diet, which consists of free access to highly palatable, energy dense, unhealthy human food, rich in carbohydrate and fat dietary components, are considered a robust model of human MetS. CAF diet-fed rats present hyperphagia, increased body weight (bw) and develop hyperinsulinaemia, hyperglycaemia and NAFLD [7,8]. In addition, the development of hypertension in animals fed a CAF diet for 10 weeks has also been reported [7]. Therefore, this diet experimental model can be especially suitable to evaluate the effectiveness of different compounds on obesity and other complications related to MetS.

Conjugated linoleic acid (CLA) is a dietary supplement that has been reported, with its effects on body weight loss being one of the most investigated [9,10]. CLA is a group of positional and geometric isomers of the omega-6 essential fatty acid linoleic acid, which present conjugated double bonds naturally produced by ruminal biohydrogenation. These double bonds can take place in different positions, generating a family of isomeric fatty acids, of which *cis*-9 (c9), *trans*-11 (t11), and *trans*-10 (t10), *cis*-12 (c12) CLA are the most naturally abundant and widely studied [11,12]. The administration of CLA to different animal species at doses from 0.5 to 1.5% of total dietary fat has been shown to reduce body fat [13,14,15,16], and a meta-analysis of human trials concluded that at an average dose of 3.2 g per day of CLA reduces body fat mass [10]. However, the results in animal studies might not be comparable with those obtained in human studies since much higher CLA doses are used in preclinical trials (27.1–81.7 g/day) than the doses in clinical trials (0.7–6.8 g/day) [10,17]. In addition, depending on the duration of CLA intake, animal species and dose of CLA, several authors have reported controversial metabolic effects of CLA consumption including insulin resistance and hepatic lipid accumulation with a consequent development of type II diabetes [18,19,20,21]. 

Therefore, the aim of this study was to evaluate the effects of a three-week supplementation of CLA on obesity and other cardiometabolic conditions associated with MetS using three low doses of CLA of similar value to that administrated to humans [22] in rats fed a CAF diet. The doses assessed in this study were 100, 200, and 300 mg CLA/kg of bw, corresponding to approximately 0.036%, 0.072%, and 0.11% in the diet, respectively. In addition, this experimental model mimics the classical model of human MetS characterised by exaggerated obesity accompanied by glucose intolerance and inflammation. Furthermore, a potential adverse effects in the liver of these animals was also evaluated by metabolomic, histological and biochemical approaches.

## 2. Materials and Methods

### 2.1. The Conjugated Linoleic Acid

CLA (Tonalin® TG 80) was purchased by BASF The Chemical Company (Düsseldorf, Germany) and is a mix of glycerides of which 80% are conjugated linoleic acids. According to the manufacturer, the product was composed of equal amounts of two CLA isomers, c9, t11 and t10, c12.

### 2.2. Experimental Procedure

**Figure 1:**
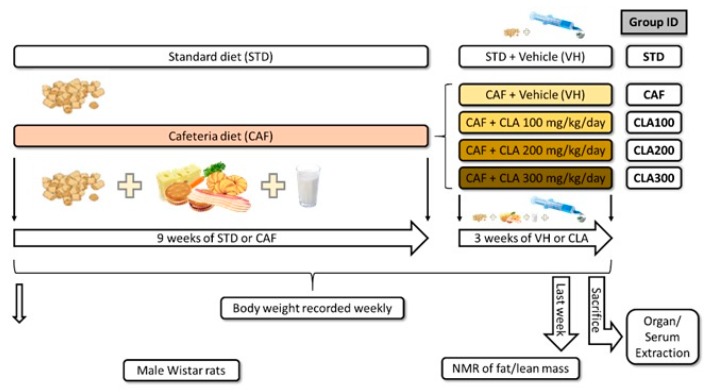
A scheme of the distribution of animals in the study. During the first nine weeks, one group was fed the standard chow diet (STD group), whereas the other group was fed the cafeteria diet (CAF group). After nine weeks, the animals were orally administered either vehicle (VH) or conjugated linoleic acid (CLA) at three doses (100, 200 and 300 mg/kg). On week twelve, the animals were sacrificed. CAF: cafeteria diet; CLA: conjugated linoleic acid; STD: standard chow diet; VH: vehicle.

Thirty male Wistar rats of 5 weeks old where purchased from Charles River Laboratories (Barcelona, Spain). The animals were maintained at 22 °C, receiving standard (STD) chow Panlab A04 (Barcelona, Spain) and tap water *ad libitum* for 10 days. Subsequently, rats were divided into two groups, STD diet-fed rats (*n* = 6), fed STD chow Panlab A04 and tap water, or CAF diet-fed rats (*n* = 24), fed fresh CAF diet in addition to STD diet and tap water. CAF diet consisted of biscuits with pate and cheese, bacon, semi-cured cheese, carrots, *ensaïmada* (traditional sweetened pastry) and milk with 20% sucrose (*w/v*) daily. As previously reported [8], the composition of the STD diet was 4% fat, 76% carbohydrates and 20% protein, whereas the CAF diet was approximately 35% fat, 51% carbohydrates and 14% protein. The different diets were administered for 12 weeks. At week 9, the STD diet-fed animals were orally administered sweetened skim condensed milk, containing 20% sucrose, as vehicle (VH) (*n* = 6 per group; STD). CAF diet-fed animals was divided into 4 groups (*n* = 6 in each group) and the animals were orally administered VH (CAF) and VH containing 100, 200 or 300 mg/kg of CLA (CLA100, CLA200 or CLA300, respectively). All doses were administered daily in a volume of 1 mL between 8:00 a.m. and 9:00 a.m. for 3 weeks. To calculate the daily consumption of CLA with respect to total food intake, both animal body weight and total food intake were determined in each animal at the beginning of CLA supplementation (week 9). The daily amount of CLA was approximately 45, 90, and 135 mg in rats weighing 450 g to obtain doses of 100, 200, and 300 mg/kg, respectively. In addition, considering that the food intake of our animals was approximately 100 g per day and that in Tonalin® TG 80 only an 80% is CLA, the percentage of CLA in diet in our study was approximately 0.036, 0.072 and 0.11%. Body weight was recorded weekly during the experiment. In addition, fat and lean mass contents were recorded by nuclear magnetic resonance (NMR) using an EchoMRI-700 (Echo Medical Systems, LLC., Texas, USA). The results are expressed as a percentage of fat or lean mass with respect to the total body weight. At the end of the experiment, rats were fasted for 3 h after the oral administration and then were sacrificed by decapitation. Serum was obtained after blood clotting and centrifugation (2000× *g*, 15 min, 4 °C) and stored at −80 °C. Fasting conditions of the animals were confirmed by evaluating the serum glucose levels with an enzymatic colorimetric assay (QCA, Barcelona, Spain) (data not shown). Livers were dissected, weighted, frozen immediately in liquid nitrogen and stored at −80 °C. The complete experimental design is schematised in Figure 1. The Animal Ethics Committee of University Rovira i Virgili approved all procedures (reference number 7959 by Generalitat de Catalunya). All of the above-mentioned experiments were carried out as authorised (European Directive 86/609/CEE and Royal Decree 223/1988 of the Spanish Ministry of Agriculture, Fisheries and Food, Madrid, Spain).

### 2.3. Biochemical and Histopathological Analyses

Serum leptin (Ref. #EZRL-83K) and insulin (Ref. #EZRMI-13K) were measured using ELISA kits (Millipore, Madrid, Spain) according to the manufacturer’s instructions. The sensitivity of the assays for leptin and insulin were 0.04 and 0.2 ng/mL, respectively. The intra-assay variations were 2.5% for leptin and 1.9% for insulin and the inter-assay variations were 3.2% for leptin and 9.2% for insulin. All samples were diluted 1:2 with assay buffer (0.05 M phosphosaline, pH 7.4, containing 0.025 M EDTA, 0.08% sodium azide and 1% BSA) and tested in duplicates. The enzyme-substrate reaction was developed using 3,3’,5,5’-tetramethylbenzidine and the optical densities were measured at 450 nm in the microtiter plate reader EON Microplate (BioTek, VT, USA). The concentrations were calculated from a standard curve obtained from eight dilutions of lyophilized native rat leptin (range 0.2–30 ng/mL) or insulin (range 0.2–10 ng/mL). GOT (glutamic oxaloacetic transaminase) and GPT (glutamate pyruvate transaminase) enzymatic activity were measured using QCA kits (Comercial Bellés, Tarragona, Spain). A piece of liver was sent for histopathology (Eldine® facilities, Tarragona, Spain). Briefly, liver pieces were thawed and fixed in 4% formaldehyde for 24 h to later undergo several dehydration steps (with ethanol at 70%, 96% and 100%, in addition to xylol/dimethyl benzene) and paraffin infiltration at 52 °C (Citaldel 2000, Thermo Scientific, Madrid, Spain). Sections of 2 µm thickness were cut (Microm HM 355S, Thermo Scientific, Madrid, Spain) and stained with haematoxylin-eosin (Varistain Gemini, Shandon, Madrid, Spain). Stained slides were analysed by a pathologist blinded to experimental groups to measure the steatosis degree, percentage of micro- and macro-steatosis, microgranulomes, lipogranulomes, portal chronic inflammation, sinusoidal dilatation and fibrosis.

### 2.4. Western Blot Analysis

Liver (60 mg) was mixed with 1 mL of RIPA (radio-immunoprecipitation assay) lysis buffer (100 mM Tri-HCl, 300 mM NaCl, 7% Tween. 10% Na-Deox, at pH 7.4) and proteases inhibitors as recommended by the TissueLyser LT protocol of Qiagen (S.G Servicios Hospitalarios, Barcelona, Spain). Samples were homogenised with the TissueLyser in 3 pulses of 15 s and 50 oscillations/s. After that, they were centrifuged at 12,000× *g* for 20 min at 4 °C and the supernatant collected and stored at −20 °C for later analysis. Quantification of the protein concentration was measured following the recommended protocol of the Pierce BCA (bicinchoninic acid) protein assay kit (Thermo Scientific, Cedex, France). Quantified samples were prepared for western blot analysis by mixing them with Bio-Rad’s Laemmli sample buffer and heated at 99 °C for 5 min. SDS-polyacrylamide gel electrophoresis (PAGE) was prepared using the TGFX Fas Cast Acrylamide Kit (Bio-Rad, Barcelona, Spain), and 25 µg of protein was loaded and run in electrophoresis buffer (25 mM Tris-Base, 1% SDS and 192 mM glycine). Proteins were transferred into PVDF (polyvinylidene difluoride) membranes using the recommended protocol by the Trans-Blot Turbo Mini PVDF Transfer packs by Bio-Rad. Blocking was performed with 5% non-fat dried milk. After that, membranes were cut around the expected location of the interested proteins and incubated with anti-rabbit p-STAT3 primary antibody (41135, Cell signalling, Barcelona, Spain) and β-Actin (A2066-100 µL, Sigma, Madrid, Spain), both diluted at 1:1000. Next, membranes were incubated with 1:10,000 diluted anti-rabbit horseradish peroxidase secondary antibody (NA9344, GE Healthcare, Barcelona, Spain) and labelled using the chemiluminescent detection reagent ECL Select (GE Healthcare, Barcelona, Spain) and GeneSys software (B:Box series, Syngene, Barcelona, Spain). Quantification by densitometry was performed with ImageJ (W.S Rasband, Bethesda, MD, USA), and the data were normalised by β-Actin.

### 2.5. Metabolite Extraction Procedure for ^1^H NMR Spectrometry

Serum extraction was performed following Bligh-Dyer procedure with slight modifications [23]. Briefly, serum (100 µL) was added to 400 µL of methanol and 100 µL of ultrapure water obtained from a Milli-Q Advantage A10 system (Madrid, Spain). After homogenisation, 200 µL of chloroform (Prolabo®, VWR, Llinars del Vallès, Spain) was added and homogenised to yield a monophasic solution. Next, an additional 600 µL of chloroform (Prolabo®, VWR, Llinars del Vallès, Spain) and 200 µL of ultrapure water were added and the samples were centrifuged at 8500× *g* for 15 min, at 4 °C, to obtain two phases separated by protein debris. The upper aqueous phase (hydrophilic metabolites) was freeze-dried and stored at −80 °C while the remaining lipidic phase (lipophilic metabolites) was dried under nitrogen stream and stored at −80 °C until further NMR measurement. Meanwhile, liver extraction was performed following the procedure described in [24] with slight modifications. Briefly, 50 mg of liver was manually homogenised using a micropestle in 1 mL of water/acetonitrile (1/1). The homogenate was centrifuged at 15,000× *g* for 30 min at 4 °C. The aqueous upper phase was separated and lyophilised overnight and, once dried, stored at −80 °C until further analysis. The lipophilic pellet was subsequently mixed with 1 mL of a solution of chloroform/methanol (2:1) at 0 °C, allowed to rest at room temperature for 10 min and then vortexed and centrifuged for 15 min at 6000× *g* at room temperature. The lipophilic supernatant was isolated from debris, dried with nitrogen flux and stored at −80 °C.

### 2.6. ^1^H NMR Spectrometry

For NMR measurements, the aqueous extracts were reconstituted in 600 μl of deuterium oxide (D_2_O) phosphate buffer (PBS (phosphate-buffered saline) 0.05 mM, pH 7.4, 99.5% D_2_O) containing 0.73 mM trisilylpropionic acid (Cortecnet®, Voisins-Le-Bretonneux, France). The dried lipophilic extracts were reconstituted with a solution of deuterated chloroform/deuterated methanol (2:1) containing 1.18 mM tetramethylsilane (TMS) and then vortexed. Both extracts were transferred into 5-mm O.D. NMR glass tubes for NMR measurement. ^1^H NMR spectra were recorded at 300 K on an Avance III 600 spectrometer (Bruker, Germany) operating at a proton frequency of 600.20 MHz using a 5-mm PABBO (proton enhanced–Smartprobe® (Bruker®) broadband gradient probe). For aqueous extracts, one-dimensional ^1^H pulse experiments were carried out using the nuclear Overhauser effect spectroscopy (NOESY) pre-saturation sequence (RD–90°–t1–90°–tm–90° ACQ) to suppress the residual water peak, and the mixing time was set at 100 ms. Solvent pre-saturation with an irradiation power of 75 Hz was applied during the recycling delay (RD = 5 s) and mixing time. The 90° pulse length was calibrated for each sample and varied from 9.95 to 10.06 µs. The spectral width was 12 kHz (20 ppm), and a total of 256 transients were collected into 64 K data points for each ^1^H spectrum. In the case of lipophilic extracts, a 90° pulse with pre-saturation sequence (zgpr) was used to supress the small residual water signal absorbed from ambient moisture by methanol. An RD of 5.0 s with acquisition time of 2.94 s were used. The 90° pulse length was calibrated for each sample and varied from 9.92 to 10.04 µs. After 4 dummy scans, a total of 128 scans were collected into 64 K data points with a spectral with of 18.6 ppm. The exponential line broadening applied before Fourier transformation was of 0.3 Hz. The frequency domain spectra were phased, baseline-corrected and referenced to the TSP or TMS signal (δ = 0 ppm) using TopSpin software (version 2.1, Bruker, Mannheim, Germany). The acquired 1H NMR were compared to references of pure compounds from the metabolic profiling AMIX spectra database (Bruker), HMDB, Chenomx NMR suite 8.4 software (Chenomx Inc., Edmonton, Canada) and databases for metabolite identification. In addition, we assigned metabolites by 1H–1H homonuclear correlation (COSY (correlation spectroscopy) and TOCSY (total correlation spectroscopy) and 1H–13C heteronuclear (HSQC) 2D NMR experiments and by correlation with pure compounds run in-house. After pre-processing, specific ^1^H NMR regions identified in the spectra were integrated and quantified using the AMIX 3.9 software package using TSP signal of buffer as internal reference [24]. A general view and metabolite assignment of a representative serum extract NMR aqueous spectra is presented in Supplementary Figure S1. 

### 2.7. Gene Expression Analysis

Liver was homogenised and RNA extraction was performed using TRIzol LS Reagent (Thermo Fisher, Madrid, Spain). Total extracted RNA was quantified and its purity measured in a Nanodrop 100 Spectrophotometer (Thermo Scientific, Madrid, Spain). RNA quality was assessed on denaturing agarose gels. Reverse transcription was performed to obtain cDNA using the High-Capacity Complementary DNA Reverse Transcription Kit (Thermo Fisher, Madrid, Spain). Quantitative PCR amplification and detection were performed in the ABI prism 7900HT real-time PCR system (Applied Biosystems) using 96-well plates and SYBR PCR Premix Reagent Ex Taq™ (Takara, Barcelona, Spain) following the commercial protocol. Relative mRNA levels of *Tnf-α* (tumour necrosis factor α), *Ccl2* (C-C motif chemokine ligand 2), *Hmgcr* (3-hydroxy-3-methyl-glutaryl-CoA reductase), *Ldlr* (low-density lipoprotein receptor), *Cyp7a1* (cytochrome P450 family 7 subfamily a member 1), *Asbt* (apical sodium-dependent bile acid transporter) and *Ppia* (peptidylprolyl isomerase A) were analysed by real-time PCR using *Ppia* as the housekeeping gene. Primer specificity was verified by melting curve analysis. The forward (FW) and reverse (RV) primers used in this study were obtained from Biomers.net (Ulm, Germany) and can be found in Supplementary Table S1. The efficiency of qPCR was calculated by evaluating a 2-fold dilution series of cDNA and calculated by E =10^(1/slope)^. The results are expressed as the logarithm of the cDNA concentration vs the obtained Ct value. The relative expression was calculated by dividing the E^Ct^ of the studied gene by the E^Ct^ of *Ppia* and then dividing by the value of the control, normalising to the STD group. Each sample was performed in triplicate.

### 2.8. Statistical Analysis

The data are expressed as the means ± standard errors of the means (SEM). Groups were compared by Student’s t-test or one-way ANOVA and Duncan’s post hoc test. Outliers were determined by Grubbs’ test. Statistical analyses were performed using XLSTAT 2017: Data Analysis and Statistical Solution for Microsoft Excel (Addinsoft, Paris, France (2017)). Graphics were prepared using GraphPad Prism 6 (GraphPad Software, San Diego, CA, USA). *p* < 0.05 was considered statistically significant.

## 3. Results

### 3.1. Body Weight Gain, Body Composition and Serum Leptin Levels

**Figure 2:**
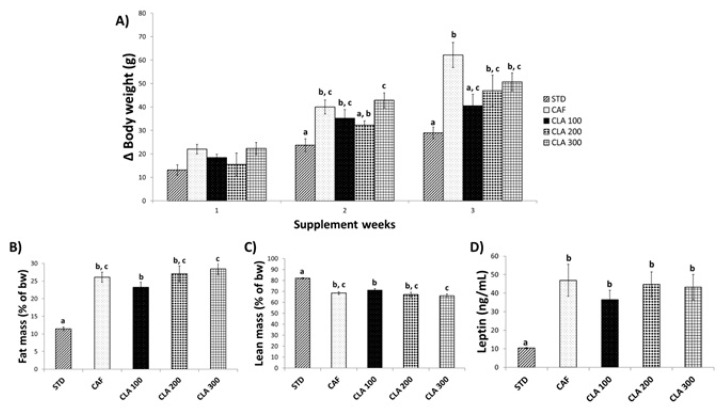
Metabolic parameters. The rats were fed the STD or CAF diet for 9 weeks and then were treated orally with CLA (100, 200 or 300 mg per kg bw) for 3 weeks. (**A**) Body weight gain (g) from the first, second and third week of the supplementation until the last day. (**B**) and (**C**) Body composition (%) assessed by NMR, including fat and lean content, respectively. (**D**) Serum levels of leptin. Data are expressed as the mean ± SEM. ^a,b,c^ denotes *p* < 0.05 assessed by one-way ANOVA and Duncan’s post hoc test. CAF: cafeteria diet; NMR: nuclear magnetic resonance; CLA: conjugated linolenic acid; STD: standard chow diet; bw: body weight.

The body weight of all animals immediately before the study was 164 ± 5 g. The STD diet-fed rats gained weight progressively during the course of the experiment. However, as expected, the weight gain in the group fed the CAF diet was significantly higher than that of the STD animals (554 ± 39 g vs 432 ± 20 g, respectively). Notably, in the last week of treatment (week 12), there was a statistically significant reduction in the body weight gain in animals supplemented with 100 mg/kg of CLA compared with that in the CAF group (Figure 2A). The effect of CLA on body weight gain was only found in the animals treated with the lowest dose of CLA and not doses of 200 and 300 mg/kg. Specific tissue weights (Supplementary Table S2) and body composition analysis indicated no statistical differences among groups, although there was a trend to decrease fat mass and increase lean mass in the group treated with CLA at a dose of 100 mg/kg (Figure 2B,C). 

A very similar pattern was also observed for the leptin levels, which exhibited a slight decrease in animals treated with the lowest dose of CLA, but the difference with respect to that in the CAF group was not statistically significant (Figure 2D). In addition, the CAF diet caused a decrease in hepatic leptin signalling compared with the STD diet measured by the quotient between p-STAT3 and serum leptin concentrations. However, no changes in this quotient were observed in the liver of CAF rats supplemented with CLA with respect to the control group (Supplementary Figure S2).

### 3.2. Glucose and Insulin Metabolism

**Figure 3:**
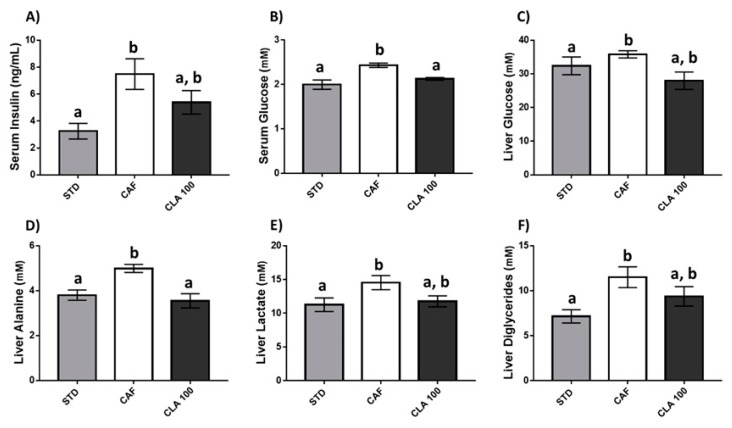
Glucose metabolism. The rats were fed the STD or CAF diet for 9 weeks and then were treated orally with CLA at 100 mg per kg of bw for 3 weeks. (**A**) Serum insulin and (**B**) serum glucose levels. The panels from (**C**–**F**) show liver metabolite levels of glucose, alanine, lactate and diglycerides, respectively. Data are expressed as the mean ± SEM. ^a,b^ denotes *p* < 0.05 assessed by one-way ANOVA and Duncan’s post hoc test. CAF: cafeteria diet; CLA: conjugated linolenic acid; STD: standard chow diet.

From here onwards, we continued our study only on the dose of 100 mg/kg as the consumption of this dose, but not doses of 200 and 300 mg/kg, exerted a significant reduction in body weight gain induced by CAF diet. In this context, the CAF diet caused an increase in both glucose and insulin values compared with levels with the STD diet. Notably, the administration of 100 mg/kg of CLA normalised them to basal levels observed in the STD group (Figure 3A,B). In addition, a very similar pattern was also observed in some individual serum metabolites identified in aqueous extract including citrate, glycerol and threonine (Table 1). At the hepatic level, animals treated with 100 mg/kg of CLA also displayed lower concentrations of glucose, alanine, lactate and diglycerides than levels in the CAF animals (Figure 3C–F), suggesting an improvement in the overall carbohydrate metabolism in animals treated with this dose of CLA. Hepatic individual concentrations of metabolites identified in the aqueous extract are shown in Table 2.

### 3.3. Serum Lipid Profile

**Figure 4:**
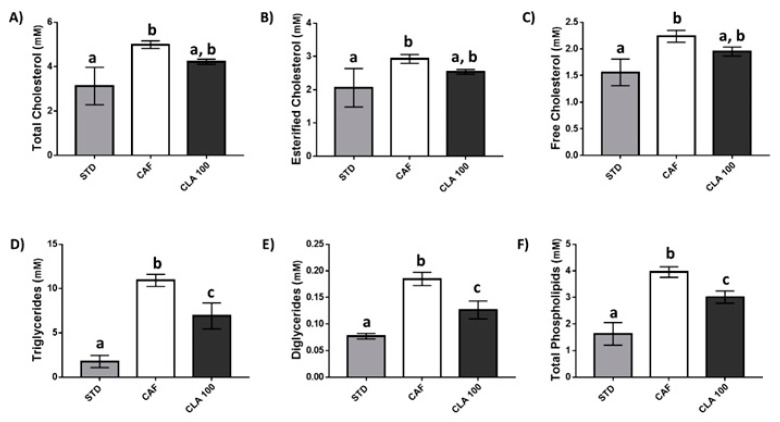
Serum lipid profile. The rats were fed the STD or CAF diet for 9 weeks and then were treated orally with CLA at 100 mg per kg of bw for 3 weeks. The panels from (**A**–**F**) show metabolite levels of different forms of cholesterol, triglycerides, diglycerides and total phospholipids, respectively. Data are expressed as the mean ± SEM. ^a,b,c^ denotes *p* < 0.05 assessed by one-way ANOVA and Duncan’s post hoc test. CAF: cafeteria diet; CLA: conjugated linolenic acid; STD: standard chow diet.

As expected, the CAF diet caused an increase in total, esterified and free cholesterol serum values compared with those in the STD diet group. However, the administration of 100 mg/kg of CLA normalised cholesterol levels in these animals to that in the STD group (Figure 4A–C). In addition, the administration of a CAF diet increased serum triglyceride, diglyceride and phospholipid concentrations compared to those of the STD group, and the administration of 100 mg/kg of CLA also produced a significant decrease in all of these values compared with those of the CAF animals (Figure 4D–F). In a similar way, the concentration of other important lipid forms including linoleic acid, oleic acid, polyunsaturated fatty acids (PUFAs), monounsaturated fatty acids (MUFAs) and docosahexaenoic acid (DHA) were reduced in the serum of CLA-treated animals with respect to the CAF group but did not reach to basal levels observed in the STD group (Table 3). 

### 3.4. Hepatic Fat Accumulation and Liver Function

**Figure 5:**
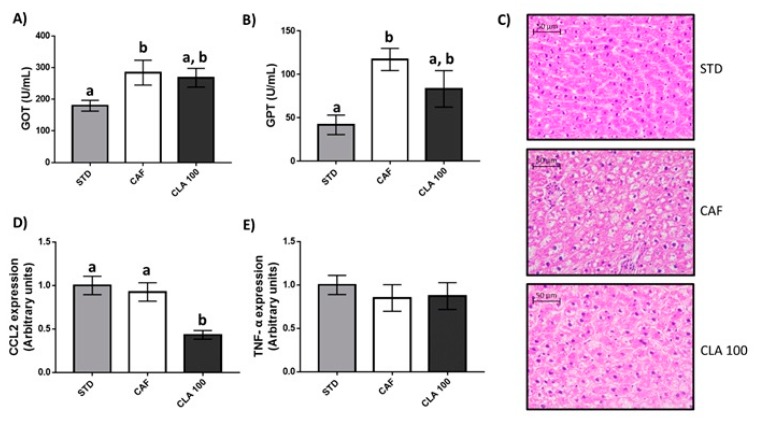
Liver function. The rats were fed the STD or CAF diet for 9 weeks and then were treated orally with CLA at 100 mg per kg of bw for 3 weeks. (**A**) GOT and (**B**) GPT serum enzymatic activities. (**C**) Representative histological sections of liver from from STD, CAF and CLA groups. Hepatic (**D**) *Ccl2* and (**E**) *Tnf-α* relative gene expression. Data are expressed as the mean ± SEM. ^a,b^ denotes *p* < 0.05 assessed by one-way ANOVA and Duncan’s post hoc test. CAF: cafeteria diet; CLA: conjugated linolenic acid; STD: standard chow diet; GOT: Glutamic oxaloacetic transaminase; GPT: glutamate pyruvate transaminase.

The CAF diet caused an increase in serum GOT and GPT enzymatic activity compared with levels in the STD group, and the administration of 100 mg/kg of CLA normalised these enzymatic activities (Figure 5A,B). In addition, CAF animals presented hepatic steatosis with respect to STD rats as indicated by the histological score of steatosis (Figure 5C). However, the administration of 100 mg/kg of CLA did not normalise the steatosis degree to basal levels and did not generate significant changes in the presence of microgranulomes and sinusoidal dilatation (Table 4). In addition, any group of animals developed portal chronic inflammation, fibrosis or lipogranulomes. In contrast, the hepatic gene expression of the inflammatory marker *Ccl2* significantly decreased after the administration of 100 mg/kg of CLA (Figure 5D). 

**Figure 6:**
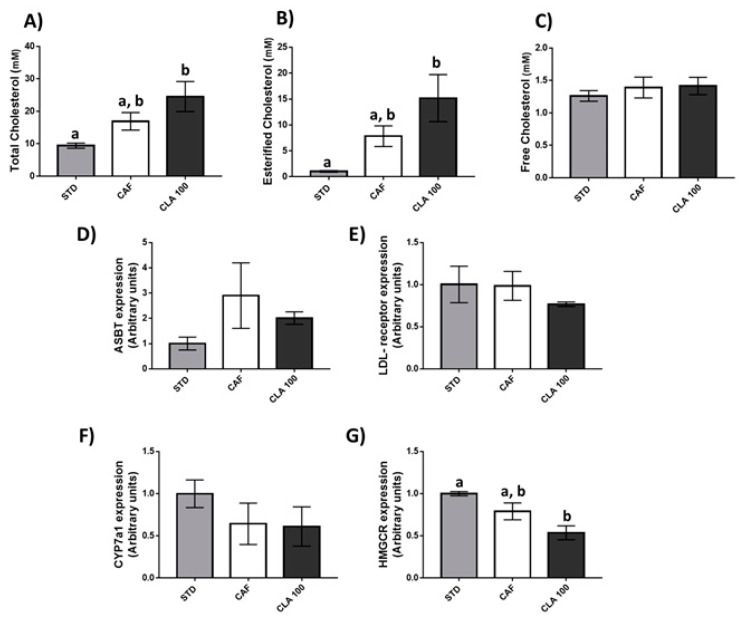
Hepatic cholesterol metabolism. The rats were fed the STD or CAF diet for 9 weeks and then were treated orally with CLA at 100 mg per kg of bw for 3 weeks. The panels from (**A**–**C**) show metabolite levels of different forms of cholesterol including total, esterified and free cholesterol, respectively. The panels from (**D**–**G**) illustrate the hepatic relative gene expression levels of *Asbt*, *Ldlr, Cyp7a1* and *Hmgcr*, respectively. Data are expressed as the mean ± SEM. ^a,b^ denotes *p* < 0.05 assessed by one-way ANOVA and Duncan’s post hoc test. CAF: cafeteria diet; CLA: conjugated linolenic acid; STD: standard chow diet.

At the metabolomics level, the administration of 100 mg/kg of CLA did not significantly change the lipid profile in the liver of these animals (Table 5), although the concentrations of total and esterified cholesterol were significantly increased with respect to the STD animals (Figure 6A,B). No changes in free cholesterol values were observed among the three groups of animals (Figure 6C). In order to understand these cholesterol alterations, the gene expression of key enzymes involved in cholesterol metabolism was studied in the liver. Accordingly, *Asbt*, *Ldlr,* and *Cyp7a1* relative gene expression was not influenced by CLA supplementation (Figure 6D–F). However, animals supplemented with CLA showed a significant downregulation in liver expression of *Hmgcr* with respect to the STD group (Figure 6G). 

## 5. Discussion

MetS is a cluster of conditions including hypertension, high blood glucose, excess body fat around the waist, and abnormal blood lipid levels, which increases the risk of cardiovascular disease and type 2 diabetes. Different bioactive compounds have been widely studied as strategies for preventing the onset of MetS and its comorbidities, with CLA supplementation being one of the most investigated for weight loss [25]. However, animal studies have mainly been carried out with diets supplemented with 0.5–1.5%, which implies a daily CLA supplementation approximately 50 times higher than those successfully used in clinical trials [10,17]. Therefore, since a greater level of inflammation, insulin resistance, and steatosis are related to the use of higher doses [26], low doses of CLA were tested in this study. Nevertheless, as the beneficial effect of body fat loss is also related to the dose of CLA used [27], the aim of this study was to test whether low doses of CLA were able to promote an ameliorating effect on obesity without inducing metabolic adverse effects. In addition, as differential effects of both CLA isomers have been reported, an equal ratio of c9,t11 and t10,c12 was used. In fact, although the isomer t10,c12 is the isomer related to the weight loss [11], the effects of this isomer on other cardiometabolic risk factors such as insulin sensitivity, markers of cardiovascular risk and liver function have raised safety concerns [28]. However, the isomer c9,t11 has not been reported to develop detrimental effects and it is generally considered safer to supplement [21], but does not have a meaningful impact on obesity [29]. This isomer has also been shown to reduce the appearance of steatosis and the concentration of inflammatory cytokines [30]. It is also known to improve mitochondrial activity in liver, modulating the release of ROS. Concretely, the c9,t11 isomer reduces ROS yield production, H_2_O_2_ concentration and promotes fatty acid oxidation rate [31]. This process, however, was reported not to be enough to compensate for the increase in fatty acid accumulation, ultimately leading to hepatic steatosis. Finally, as CAF diet-fed rats present not only obesity, but also other cardiometabolic risk factors, the impact of low doses of an equal ratio of c9,t11 and t10,c12 isomers was also investigated in other abnormalities associated with MetS. 

Our results showed that supplementation with low doses of CLA caused a decrease in the body weight gain in the animals fed the CAF diet. The doses assessed in this study (100, 200 and 300 mg CLA/kg), correspond to approximately 0.036, 0.072 and 0.11% in the diet, respectively, and are similar to the human recommended doses [22]. Paradoxically, the most effective dose to reduce the body weight gain at the end of the study was the lowest dose. Supporting these results, mice supplemented with moderate doses of CLA (150 and 500 mg/kg) also showed a decrease in body weight gain, which was not greater for the higher dose [32]. Interestingly, the changes in body weight gain observed after the lowest dose of CLA supplementation in our study were not accompanied by a reduction in body fat mass, nor by a reduction in lean mass, which is the major undesirable effect obtained when a caloric restriction diet is used to reduce body weight [33]. In addition, as expected, the CAF group showed increased serum leptin levels reinforcing the well-established association between leptin resistance and obesity. However, CLA supplementation failed to reduce the concentration of this hormone as well as to restore the hepatic leptin sensitivity in these animals, indicating that the anti-obesity effect of CLA is independent of the leptin system. 

In this study, CAF diet-fed rats showed elevated serum glucose and insulin levels. The presence of peripheral insulin resistance and increased pancreatic insulin secretion have been reported in this animal model [34]. Nevertheless, despite insulin resistance being one of the most commonly reported detrimental effects of CLA supplementation, the results of this study not only demonstrated no adverse effects on insulin signalling but that the use of a low dose of CLA improved the overall carbohydrate metabolism in these animals. In this sense, serum and liver glucose and circulating insulin were normalised to STD levels after CLA supplementation, counteracting the effect of the CAF diet. In addition, a reduction in both alanine and lactate hepatic levels was found in animals supplemented with CLA, reaching values similar to those of the STD animals. Reduced levels of these hepatic metabolites, namely, alanine [35] and lactate [36], might indicate a lower gluconeogenesis that would result in better insulin signalling because the liver would not need to produce as much glucose. However, a direct confirmation of this hypothesis should be further tested in future studies. In addition, isoleucine and other BCAAs have also been directly linked with the development of type II diabetes [37,38], and our results showed a reduction in hepatic isoleucine in animals supplemented with a low dose of CLA. Increased hepatic diglyceride levels have also been associated with insulin resistance because diglycerides modulate the affinity of the insulin receptor, through protein kinase Ce (PKCe) [39,40,41], and our results showed that a low dose of CLA significantly reduced their concentrations. The effect of low-dose CLA supplementation on lipid metabolism was also examined in this study. Our results showed an extensive reduction in several lipid metabolite concentrations, suggesting an improvement in the management of fat in CLA-supplemented animals. Supporting these results, an improvement in the lipid profile caused by CLA supplementation has been previously reported in animal [42] and human studies [43]. In fact, diverse lines of research indicate that the CLA effects on lipid metabolism are mediated by the modulation of eicosanoid formation [44], and these effect seems to be more favourably influenced by the c9,t11 isomers than others in both animals [45] and humans [46].

NAFLD is considered as one of the main harmful concomitant outcomes from CLA supplementation [21]. Factors leading to hepatic lipid accumulation are multifactorial and involve increased fatty acid influx and synthesis, as well as altered fatty acid oxidation and triglyceride secretion [47,48,49,50,51,52,53]. In this sense, a decrease in the PUFA/MUFA ratio has been considered as a marker of liver steatosis in different metabolomics studies [24]. However, our results indicated that CLA consumption did not reduce this ratio with respect to CAF animals. In addition, the hepatic histopathological analyses performed in our animals showed no signs of liver damage aggravation in rats supplemented with CLA with respect to CAF animals, and even a lower steatosis degree was observed in animals supplemented with CLA. Supporting these results, serum GOT and GPT enzymatic activities were also normalised in the CLA group with respect to the STD group, suggesting a slight recovery of the hepatic damage associated with the CAF diet. In addition, many CLA supplements are currently formulated to contain an equal ratio of c9,t11 and t10,c12, aiming to obtain the reduction of fat mass and obesity, but compensating with the health benefits of the c9,t11 isomer. This slight improvement of hepatic steatosis by the c9,t11 isomer, besides the low dose of t10,c12, could explain why we did not find signs of worsening liver status, as c9,t11 might be able to compensate the damage by the t10,c12. However, it is difficult to assess and compare among experiments as doses and diet play a major role in the effect of CLA.

Conversely, a significant increase in the hepatic esterified cholesterol content after CLA supplementation was observed in these animals. To uncover the potential mechanism of the hepatic cholesterol-increasing effect of CLA, we investigated the relative gene expression of *Hmgcr*, *Asbt*, *Ldlr*, and *Cyp7a1* that are the key regulators associated with cholesterol metabolism and have been reported to be regulated by dietary intervention [54]. However, our data demonstrated that this increase was not related to changes in hepatic cholesterol synthesis, cholesterol conversion into bile acids, cholesterol caption or hepatic bile acid reabsorption. Thus, further research is worthy to be conducted assess the effects of dietary supplemental CLA on hepatic cholesterol deposition and elucidating its possible mechanism.

Finally, the role of CLA as pro- or anti-inflammatory agent has no clear consensus as of yet among the scientific community. While several studies in rodents and cell culture showed CLA is an anti-inflammatory molecule, some authors do not consider that its anti-inflammatory effects have been sufficiently demonstrated in humans [55]. In addition, other studies have clearly marked CLA as a proinflammatory inductor [56,57], indicating that this effect could be greatly heterogeneous depending on physiological conditions, animal species and the dose of CLA. In addition, initial studies in cells showed that CLA, specifically t10,c12, presented ameliorating effects over the production of reactive oxygen species (ROS), correlating to lower gene expression of inflammatory markers [58]. Some studies in humans revealed consistent improvements in inflammation with a reduction in TNF-α (tumour necrosis factor α) and IL-1β (interleukin 1-β) levels [59], while others found increases in TNF-α values [56]. Thus, although there is no clear consensus of the effect of CLA on inflammation, our results indicated that low doses of CLA have the ability to decrease the gene expression of *Ccl2* in the liver as well as the levels of diglycerides, which have been reported as key markers of hepatic inflammation [60,61]. In addition, the levels of niacinamide were also significantly reduced in CLA-supplemented animals, indicating the ability of these animals to control oxidative stress and, consequently, to reduce ROS-induced damage in this tissue [62]. However, non-significant changes in *Tnf-α* mRNA levels were observed and, therefore, further studies are warranted in order to determine the potential anti-inflammatory effect of low-doses of CLA.

Finally, in this study, the most effective dose of CLA for reducing body fat was 100 mg/kg/day. This dose, using a translation of animal to human doses [63] and estimating the daily intake for an 80 kg human, correspond to an intake of 2 g/day. Although experimental results obtained in our study cannot be directly translatable to humans, the fact that only 100 mg/kg of CLA ameliorate the metabolic alterations induced by a CAF diet suggests that the utilization of CLA in the diets of obese humans could be a good strategy for improving their health outcomes.

## 6. Conclusions

In summary, long-term supplementation with CLA at a dose of 100 mg/kg caused a decrease in the body weight gain in animals fed a CAF diet. In addition, other important cardiometabolic conditions associated with MetS were also improved after CLA supplementation. In particular, an improvement in glucose and lipid metabolism was observed. Notably, although no major detrimental effect at this dose of CLA was found in our study, a note of caution should be sounded concerning the increased cholesterol values observed in the liver and, thus, further studies are needed to elucidate the molecular mechanisms involved in this phenomenon. In addition, these results corroborated with other studies carried out in humans in which 3 g of CLA (a slightly higher dose than the one we have studied) was given to normal weight, overweight and obese subjects for up to six months and no adverse effects on insulin sensitivity, blood glucose and liver function were reported [22]. The use of a CLA mixture with equal ratios of both isomers used in this study could also be decisive in this study. However, as the preconditions of our experiment are different from others reported, as we use the CAF diet as an inducer of MetS, we cannot rule out that this lack of detrimental qualities of CLA might not be due to a different metabolic and physiological status. Studies with rats have been performed using high-fat diets, inducing different metabolic alterations in which CLA might have a different impact. A synergic effect coming from the low dose of CLA, with a better experimental model that mimicked human MetS, might be the reason why we are not finding signs of inflammation, insulin resistance and NAFLD but significant beneficial effects on MetS. Thus, our results suggest that long-term supplementation with low-dose CLA might have a deeper, healthy impact on obesity.

## Figures and Tables

**Table 1:** Serum individual concentrations (mM) of metabolites identified in aqueous extracts.

	STD	CAF	CLA 100
**O-Acetylcarnitine**	0.113 ± 0.01	0.119 ± 0.01	0.11 ± 0.01
**Formate**	0.42 ± 0.03	0.42 ± 0.01	0.46 ± 0.02
**Glycerol**	1.32 ± 0.1 ^a^	1.71 ± 0.1 ^b^	1.52 ± 0.2 ^a,b^
**Acetate**	3.52 ± 0.1	3.65 ± 0.1	3.77 ± 0.1
**3-Hidroxybutyrate**	1.25 ± 0.2	0.87 ± 0.1	1.21 ± 0.3
**Glucose**	2.00 ± 0.11 ^a^	2.43 ± 0.05 ^b^	2.13 ± 0.03 ^a^
**Piruvate**	0.44 ± 0.1	0.64 ± 0.1	0.6 ± 0.1
**Succinate**	0.19 ± 0.01	0.17 ± 0.01	0.2 ± 0.02
**Lactate**	20.13 ± 2.7	24.56 ± 3.1	24.14 ± 3.2
**Citrate**	0.17 ± 0.01 ^a^	0.22 ± 0.01 ^b^	0.18 ± 0.01 ^a^
**Asparagine**	0.46 ± 0.02	0.51 ± 0.04	0.53 ± 0.02
**Leucine**	0.97 ± 0.02	0.94 ± 0.08	0.96 ± 0.04
**Threonine**	1.35 ± 0.1 ^a^	1.67 ± 0.1 ^b^	1.57 ± 0.1 ^a,b^
**Tryptophan**	0.74 ± 0.04	0.87 ± 0.06	0.8 ± 0.05
**Tyrosine**	0.61 ± 0.04	0.76 ± 0.07	0.65 ± 0.03
**Proline**	0.89 ± 0.1	1.14 ± 0.1	1.04 ± 0.1
**Isoleucine**	0.68 ± 0.02	0.62 ± 0.05	0.6 ± 0.03
**Glycine**	1.9 ± 0.1	1.57 ± 0.1	1.64 ± 0.13
**Glutamate**	1.01 ± 0.05	1.11 ± 0.07	1.11 ± 0.04
**Glutamine**	5.52 ± 0.1	5.51 ± 0.4	5.39 ± 0.1
**Methionine**	0.61 ± 0.01	0.69 ± 0.04	0.61 ± 0.01
**Lysine**	2.3 ± 0.1	2.45 ± 0.2	2.57 ± 0.2
**Valine**	1.11 ± 0.1	1.17 ± 0.1	1.11 ± 0.1
**Serine**	2.2 ± 0.1 ^a^	2.6 ± 0.2 ^a,b^	2.75 ± 0.1 ^b^
**Alanine**	3.06 ± 0.1	3.99 ± 0.5	3.54 ± 0.2
**Phenylalanine**	0.56 ± 0.01	0.61 ± 0.03	0.55 ± 0.01
**Taurine**	0.29 ± 0.02	0.29 ± 0.01	0.31 ± 0.01
**Carnosine**	0.26 ± 0.01	0.23 ± 0.03	0.22 ± 0.01
**Choline**	0.16 ± 0.005 ^a^	0.14 ± 0.008 ^b^	0.14 ± 0.004 ^b^
**Betaine**	0.82 ± 0.07 ^a^	0.54 ± 0.04 ^b^	0.58 ± 0.03 ^b^
**Creatinine**	0.09 ± 0.003	0.08 ± 0.007	0.09 ± 0.003
**Glutathione**	0.04 ± 0.01	0.05 ± 0.01	0.05 ± 0.01
**Allantoin**	0.24 ± 0.01 ^a^	0.19 ± 0.001 ^b^	0.2 ± 0.01 ^b^
**Creatine**	1.95 ± 0.2	2.57 ± 0.2	2.35 ± 0.2
**Creatine phosphate**	0.3 ± 0.01	0.33 ± 0.03	0.27 ± 0.01
**Pantothenate**	0.06 ± 0.002	0.07 ± 0.01	0.05 ± 0.007

Values are presented as the mean ± SEM. ^a,b^ denotes *p* < 0.05 assessed by one-way ANOVA and Duncan’s post hoc test. CAF: cafeteria diet; CLA: conjugated linolenic acid; STD: standard chow diet.

**Table 2:** Hepatic individual concentrations (mM) of metabolites identified in aqueous extracts.

	STD	CAF	CLA 100
**Valine**	0.48 ± 0.02	0.57 ± 0.04	0.49 ± 0.04
**Isoleucine**	0.3 ± 0.01 ^a^	0.27 ± 0.02 ^a,b^	0.22 ± 0.01 ^b^
**Leucine**	1.17 ± 0.1	1.18 ± 0.1	1.04 ± 0.1
**Glycine**	0.1 ± 0.016	0.1 ± 0.007	0.09 ± 0.015
**Alanine**	3.8 ± 0.2 ^a^	4.99 ± 0.2 ^b^	3.54 ± 0.3 ^a^
**Glutamine**	5.51 ± 0.4	4.47 ± 0.4	5.13 ± 0.3
**Tyrosine**	0.27 ± 0.009 ^a^	0.26 ± 0.015 ^a^	0.2 ± 0.014 ^b^
**Histidine**	0.61 ± 0.03	0.57 ± 0.03	0.53 ± 0.02
**Methionine**	0.33 ± 0.01 ^a^	0.25 ± 0.01 ^b^	0.23 ± 0.01 ^b^
**Glutamate**	2.26 ± 0.1	2.89 ± 0.2	2.41 ± 0.3
**Phenylalanine**	0.8 ± 0.05	0.84 ± 0.07	0.7 ± 0.04
**Glucose**	32.38 ± 2.6 ^a^	35.79 ± 1.0 ^b^	27.95 ± 2.6 ^a,b^
**Succinate**	1.5 ± 0.1	1.23 ± 0.5	1.54 ± 0.3
**Acetate**	0.37 ± 0.02	0.47 ± 0.05	0.5 ± 0.13
**Lactate**	11.26 ± 1.0 ^a^	14.54 ± 1.0 ^b^	11.76 ± 0.8 ^a,b^
**Fumarate**	0.09 ± 0.009	0.07 ± 0.008	0.1 ± 0.01
**NAD^+^**	0.64 ± 0.04	0.54 ± 0.1	0.64 ± 0.03
**NADP^+^**	0.37 ± 0.03	0.32 ± 0.03	0.34 ± 0.02
**3-Hydroxybutyrate**	0.47 ± 0.05	0.35 ± 0.02	0.41 ± 0.05
**Uridine**	0.55 ± 0.02	0.56 ± 0.09	0.43 ± 0.02
**Choline**	0.12 ± 0.005 ^a^	0.09 ± 0.005 ^a,b^	0.08 ± 0.016 ^b^
**Phosphocholine**	1.48 ± 0.1	1.13 ± 0.2	1.05 ± 0.3
**Beatine**	2.32 ± 0.2 ^a^	1.08 ± 0.1^b^	1.04 ± 0.2 ^b^
**Glutathione**	4.07 ± 0.8	2.69 ± 0.4	3.15 ± 0.6
**Niacinamide**	0.3 ± 0.02 ^a^	0.31 ± 0.01 ^a^	0.22 ± 0.01 ^b^
**Ascorbate**	1.46 ± 0.1	1.52 ± 0.22	1.6 ± 0.1
**Dimethylamine**	0.04 ± 0.003 ^a^	0.03 ± 0.001 ^a,b^	0.02 ± 0.002 ^b^
**Inosine**	1.94 ± 0.1	1.98 ± 0.2	1.59 ± 0.1
**Creatinine**	0.86 ± 0.2	0.59 ± 0.1	0.44 ± 0.1
**Creatine phosphate**	1.86 ± 0.1	2.00 ± 0.3	1.59 ± 0.1
**Creatine**	0.09 ± 0.005	0.09 ± 0.01	0.08 ± 0.01

Values are presented as the mean ± SEM. ^a,b^ denotes *p* < 0.05 assessed by one-way ANOVA and Duncan’s post hoc test. CAF: cafeteria diet; CLA: conjugated linolenic acid; STD: standard chow diet; NAD^+^: nicotinamide adenine dinucleotide; NADP^+^: nicotinamide adenine dinucleotide phosphate.

**Table 3:** Serum individual concentrations (mM) of metabolites identified in lipophilic extracts.

	STD	CAF	CLA 100
**Total Cholesterol**	3.12 ± 0.8 ^a^	4.98 ± 0.1 ^b^	4.12 ± 0.1 ^a,b^
**Free Cholesterol**	1.55 ± 0.2 ^a^	2.23 ± 0.1 ^b^	1.94 ± 0.1 ^a,b^
**Esterified Cholesterol**	2.05 ± 0.5 ^a^	2.92 ± 0.1 ^b^	2.53 ± 0.1 ^a,b^
**Triglycerides**	1.76 ± 0.6 ^a^	10.91 ± 0.6 ^b^	6.89 ± 1.4 ^c^
**Diglycerides**	0.07 ± 0.01 ^a^	0.18 ± 0.01 ^b^	0.12 ± 0.02 ^c^
**Total Phospholipids**	1.62 ± 0.4 ^a^	3.95 ± 0.1 ^b^	3.01 ± 0.2 ^c^
**Linoleic acid**	1.25 ± 0.4 ^a^	3.42 ± 0.1 ^b^	2.09 ± 0.1 ^c^
**Oleic acid**	1.71 ± 0.6 ^a^	14.33 ± 0.7 ^b^	8.88 ± 2 ^c^
**Sphingomyelin**	0.51 ± 0.03	0.48 ± 0.01	0.49 ± 0.02
**ARA + EPA**	1.97 ± 0.5	2.22 ± 0.2	1.79 ± 0.1
**DHA**	0.13 ± 0.03 ^a^	0.26 ± 0.02 ^b^	0.2 ± 0.01 ^a,b^
**Omega-3**	0.6 ± 0.14 ^a^	0.98 ± 0.05 ^b^	0.75 ± 0.02 ^b^
**Phosphocholine**	2.84 ± 0.7 ^a^	5.83 ± 0.2 ^b^	4.63 ± 0.2 ^b^
**PUFA**	6.84 ± 1.8	9.56 ± 0.8	7.6 ± 0.2
**MUFA**	4.94 ± 1.5 ^a^	21.24 ± 1 ^b^	13.12 ± 2.4 ^c^

Values are presented as the mean ± SEM. ^a,b,c^ denotes *p* < 0.05 assessed by one-way ANOVA and Duncan’s post hoc test. CAF: cafeteria diet; CLA: conjugated linolenic acid; STD: standard chow diet; ARA: arachidonic acid; EPA: eicosapentaenoic acid; DHA: docosahexaenoic acid; PUFA: polyunsaturated fatty acid; MUFA: monounsaturated fatty acid.

**Table 4:** Summary of liver histological analysis.

	STD	CAF	CLA 100
**Steatosis degree** (0 to 3 in severity)	0.67 ± 0.3 ^a^	1.50 ± 0.2 ^b^	1.33 ± 0.2 ^a,b^
**Sinusoidal dilatation** (0 to 2 in severity)	0.17 ± 0.2	0.33 ± 0.2	0.67 ± 0.2
**Microgranulomes** (number of samples)	2/6	1/6	2/6
**Fibrosis degree** (0 to 4 in severity)	0	0	0
**Portal inflammation** (0 to 2 in severity)	0	0	0
**Lipogranulomes** (number of samples)	0/6	0/6	0/6

Values are presented as the mean ± SEM. ^a,b^ denotes *p* < 0.05 assessed by one-way ANOVA and Duncan’s post hoc test.

**Table 5:** Summary of metabolites from the liver lipidic extraction.

	STD	CAF	CLA 100
**Total Cholesterol**	9.42 ± 0.7 ^a^	16.91 ± 2.6 ^a,b^	24.53 ± 4.6 ^b^
**Free Cholesterol**	1.26 ± 0.08	1.38 ± 0.16	1.41 ± 0.13
**Esterified Cholesterol**	1.05 ± 0.1 ^a^	7.83 ± 1.9 ^a,b^	15.17 ± 4.5 ^b^
**Triglycerides**	42.02 ± 3.5 ^a^	81.03 ± 11.3 ^b^	87.01 ± 11.1 ^b^
**Diglycerides**	7.14 ± 0.7 ^a^	11.51 ± 1.1 ^b^	9.37 ± 1 ^a,b^
**Sphingomyelin**	2.88 ± 0.1	2.76 ± 0.4	2.84 ± 0.3
**ARA + EPA**	28.66 ± 2.3	25.02 ± 3.5	25.61 ± 2
**Plasmalogen**	1.36 ± 0.1 ^a^	1.62 ± 0.2 ^a^	2.58 ± 0.3 ^b^
**Total Phospholipids**	48.1 ± 5.5	45.42 ± 7.4	45.34 ± 5.5
**Phosphoethanolamine**	17.37 ± 3.1	20.32 ± 2.6	20.68 ± 1.7
**Linoleic acid**	32.29 ± 2.8	36.05 ± 6.1	44.94 ± 5.7
**Oleic acid**	16.96 ± 2.4 ^a^	86.78 ± 14.6 ^b^	87 ± 16.3 ^b^
**Omega-3**	0.05 ± 0.002 ^a^	0.03 ± 0.004 ^b^	0.03 ± 0.002 ^b^
**DHA**	4.19 ± 0.6	4.22 ± 0.8	4.64 ± 0.2
**PUFA**	120.63 ± 10.5	112.11 ± 15.6	118.57 ± 7.1
**MUFA**	38.89 ± 4.5 ^a^	109.45 ± 19.6 ^b^	120.16 ± 23.7 ^b^
**Total fatty acids**	150.44 ± 9.8 ^a^	277.54 ± 35.5 ^b^	290.55 ± 9.8 ^b^

Values are presented as the mean ± SEM. ^a,b^ denotes *p* < 0.05 assessed by one-way ANOVA and Duncan’s post hoc test. CAF: cafeteria diet; CLA: conjugated linolenic acid; STD: standard chow diet; ARA: arachidonic acid; EPA: eicosapentaenoic acid; DHA: docosahexaenoic acid; PUFA: polyunsaturated fatty acid; MUFA: monounsaturated fatty acid.

## Supplementary Materials

The following are available online at https://www.mdpi.com/2072-6643/12/2/408/s1, Table S1: A summary of the rat-specific primer sequences used for qPCR analysis, Table S2: Relative tissue weights, Figure S1: General view and metabolite assignment of representative serum extract NMR aqueous spectra, Figure S2: Hepatic leptin sensitivity.
