# Supplementary material for: Beneficial Effects of a Low-dose of Conjugated Linoleic Acid on Body Weight Gain and other Cardiometabolic Risk Factors in Cafeteria Diet-fed Rats
Source: Nutrients. 2020 Feb 4;12(2):408. doi: 10.3390/nu12020408 (PMC7071287; doi:10.3390/nu12020408)
Supplement: Supplementary file 1 [file nutrients-12-00408-s001.pdf]

Supplemental data for:

Beneficial Effects of a Low-dose of Conjugated Linoleic Acid on Body Weight Gain and Other Cardiometabolic Risk Factors in Cafeteria Diet-fed Rats

Miguel Martín-González [1,#], Héctor Palacios [1,2,#], Miguel A Rodríguez [2], Lluís Arola [1,3], Gerard Aragonès [1,*] and Begoña Muguerza [1]

[1] Universitat Rovira i Virgili, Department of Biochemistry and Biotechnology, Nutrigenomics Research Group, Tarragona, Spain.

[2] Eurecat, Centre Tecnològic de Catalunya, Centre for Omic Sciences (COS), Joint Unit Universitat Rovira i Virgili-EURECAT, Unique Scientific and Technical Infrastructures (ICTS), Reus, Spain.

[3] Eurecat, Centre Tecnològic de Catalunya, Biotechnological Area, Reus, Spain.

# Both authors contributed equally to this manuscript

[*] Correspondence: gerard.aragones@urv.cat; Tel.: +34-977-558-188

Table S1. Primer sequences used in qPCR amplification.

Gene	Sequence	Product size (bp)	GenBank accession nº
Ccl2	Forward 5′-GCTGCTACTCATTCACTGGC<br>Reverse 5′-GGTGCTGAAGTCCTTAGGGT	241	NM_031530.1
Tnf-α	Forward 5′- ACCACGCTCTTCTGTCTACTG<br>Reverse 5′ - CTTGGTGGTTTGCTACGAC	169	NM_012675.3
Ppia	Forward 5′ - CTTCGAGCTGTTTGCAGACAA<br>Reverse 5′- AAGTCACCACCCTGGCACAT	138	NM_017101.1
Hmgcr	Forward 5′ - CCTGGTTCTTGTTCACGCTC<br>Reverse 5′ - GCTCGATGTCCATGCTGATC	210	NM_013134.2
Ldlr	Forward 5′ - AGTGCGATGGCCCTAACAAA<br>Reverse 5′ - CTCGTTGGTCTTGCACTCCT	128	NM_175762.2
Cyp7a1	Forward 5′ - GTTGATTCCGTACCTGGGCT<br>Reverse 5′ - TGCTTCTGTGTCCAAATGCC	230	NM_012942.2
Asbt	Forward 5′ - TGGGCTTCCTCTGTCAGTTT<br>Reverse 5′ - GCAAAGGGGCATCATTCCAA	224	NM_017222.2

Ccl2, C-C motif chemokine ligand 2; *Tnf-α*, tumour necrosis factor α; *Ppia*, peptidylprolyl isomerase A; *Hmgcr* reductase, 3-hidroxi-3-metil-glutaril-CoA reductase; *Ldlr*, Low-Density Lipoprotein receptor; *Cyp7a1*, Cytochrome P450 Family 7 Subfamily A Member 1; *Asbt*, Apical Sodium Dependent Bile Acid Transporter.

Table S2. Relative tissue weights.

	STD	CAF	CLA 100	CLA 200	CLA 300
**Liver (%)**	2.92 ± 0.1	2.98 ± 0.1	2.94 ± 0.1	3.00 ± 0.1	2.80 ± 0.1
**Muscle (%)**	0.59 ± 0.01^{a}	0.45 ± 0.03^{b}	0.49 ± 0.02^{b}	0.48 ± 0.02^{b}	0.44 ± 0.01^{b}
**eWAT (%)**	2.03 ± 0.2^{a}	3.61 ± 0.3^{b}	3.73 ± 0.4$^{b, c}$	3.68 ± 0.3$^{b, c}$	4.57 ± 0.1^{c}
**iWAT (%)**	0.23 ± 0.03^{a}	1.03 ± 0.2^{b}	0.68 ± 0.2$^{a, b}$	1.22 ± 0.4^{b}	1.34 ± 0.3^{b}
**rWAT (%)**	0.83 ± 0.04^{a}	1.72 ± 0.2^{b}	1.77 ± 0.2^{b}	1.75 ± 0.2^{b}	2.02 ± 0.2^{b}
**BAT (%)**	0.12 ± 0.01^{a}	0.22 ± 0.02^{c}	0.20 ± 0.01$^{b, c}$	0.18 ± 0.01^{b}	0.22 ± 0.01^{c}

BAT, brown adipose tissue; Bw, body weight; eWAT, iWAT and rWAT, epididymal, inguinal and retroperitoneal white adipose tissue, respectively. Values are presented as the mean ± SEM of six animals per group. Different letters represent significant differences between groups (one-way ANOVA and Duncan's post hoc test; $P<0·05$).

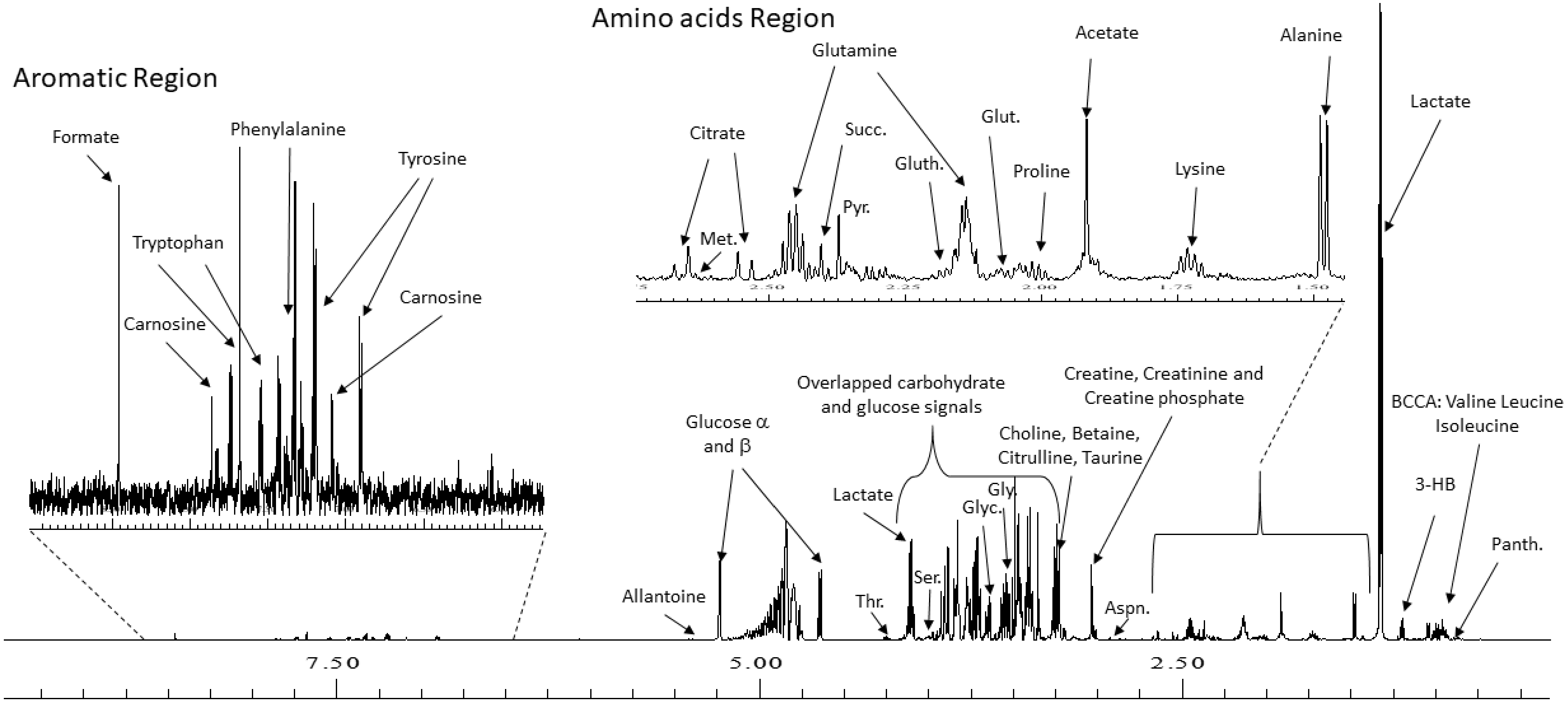

Figure S1. General view and metabolite assignment of representative serum extract NMR aqueous spectra. BCCA: Branched Chain Aminoacids; 3-HB: 3-Hydroxybutirate; Panth: Panthotenate; Glut: Glutamate; Gluth: Glutathione; Aspn: Asparagine; Pyr: Pyruvate; Succ: Succinate; Met: Methionine; Gly: Glycine; Glyc: Glycerol; Ser: Serine; Thr: Threonine;.

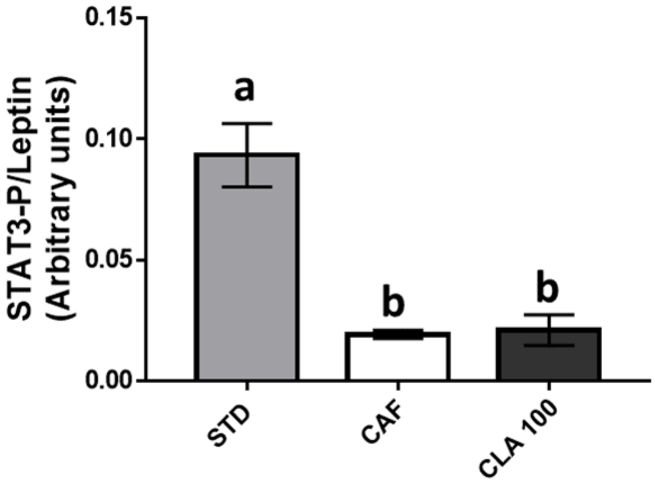

Figure S2. Hepatic leptin sensitivity. Leptin sensitivity values was obtained by the ratio between p-STAT3 (assessed by western blotting) and serum leptin levels as previously described [1]. The rats were fed the STD or CAF diet for 9 weeks and then were treated orally with CLA at 100 mg per kg of bw for 3 weeks. Data are expressed as the mean ± SEM. ^{a,b} denotes $p < 0.05$ assessed by one-way ANOVA and Duncan's post hoc test. CAF: cafeteria diet; CLA: conjugated linolenic acid; STD: standard chow diet.

[1] Ardid-Ruiz A, Ibars M, Mena P, Del Rio D, Muguerza B, Bladé C, Arola L, Aragonès G, Suárez M. Potential Involvement of Peripheral Leptin/STAT3 Signaling in the Effects of Resveratrol and Its Metabolites on Reducing Body Fat Accumulation. *Nutrients*. **2018,** *10*, E1757
